# Importation of Ticks on Companion Animals and the Risk of Spread of Tick-Borne Diseases to Non-Endemic Regions in Europe

**DOI:** 10.3390/ani11010006

**Published:** 2020-12-22

**Authors:** Alicja Buczek, Weronika Buczek

**Affiliations:** Chair and Department of Biology and Parasitology, Faculty of Health Sciences, Medical University of Lublin, 20-080 Lublin, Poland; wera1301@gmail.com

**Keywords:** ticks, companion animals, importation of ticks, ticks on travelling dogs, transmission of tick-borne pathogens

## Abstract

**Simple Summary:**

The risk of transmission of pathogen-infected ticks by dogs and cats transported by humans has increased substantially in recent decades due to the rise in tourist and economic migration rates. Therefore, we highlight the role of companion animals, mainly dogs, travelling with their owners or importation of these animals in the transmission of ticks and tick-borne diseases to non-endemic areas. The brown dog tick *Rhipicephalus sanguineus*, which is a vector of numerous pathogens causing diseases in animals and humans, is imported most frequently to many European countries from endemic areas in the Mediterranean region or from other parts of the world. Additionally, alien tick species with high epizootic and epidemiological importance can be imported while attached to dog skin to Europe from other continents. Companion animals play an even greater role in the spread of autochthonous tick species and transmission of pathogens to other animals and humans. Before travelling to endemic areas of tick-borne diseases, tourists should be acquainted with prophylaxis methods to protect themselves and their companion animals against tick attacks.

**Abstract:**

Increased human mobility elevates the risk of exposure of companion animals travelling with their owners or imported from other regions to tick attacks. In this study, we highlight the potential role of dogs and cats taken for tourist trips or imported animals in the spread of ticks and tick-borne pathogens. The *Rhipicephalus sanguineus* tick, which is a vector of numerous pathogens causing diseases in animals and humans, is imported most frequently from endemic areas to many European countries. Additionally, alien tick species with high epizootic and epidemiological importance can be imported on dogs from other continents. Companion animals play an even greater role in the spread of autochthonous tick species and transmission of tick pathogens to other animals and humans. Although the veterinary and medical effects of the parasitism of ticks carried by companion animals travelling with owners or imported animals are poorly assessed, these animals seem to play a role in the rapid spread of tick-borne diseases. Development of strategies for protection of the health of companion animals in different geographic regions should take into account the potential emergence of unknown animal tick-borne diseases that can be transmitted by imported ticks.

## 1. Introduction

Companion animals, such as dogs (*Canis lupus familiaris*) and cats (*Felis catus*), can be hosts for various tick species from the family Ixodidae [1,2,3,4,5,6,7,8,9]. In Europe, these animals are parasitized by tick species from the genera *Dermacentor, Haemaphysalis, Hyalomma, Rhipicephalus*, and *Ixodes* [10,11]. In the northern, western, and central parts of the continent, companion animals are most often attacked by the sheep tick *Ixodes ricinus*, the hedgehog tick *Ix. hexagonus*, the dog tick *Ix. canisuga*, and the meadow tick *Dermacentor reticulatus*. The most frequent attacks in the north-eastern part of Europe are ascribed to the taiga tick *Ix. persulcatus*, whereas the brown dog tick *Rhipicephalus sanguineus* and *Rh. turanicus* have been reported to infest animals primarily in the southern part of Europe. Other tick species (e.g., the red sheep tick *Haemaphysalis punctata*, the rodent tick *Ha. concinna,* the ornate sheep tick *De. marginatus*, and the southern rodent tick *Ix. acuminatus)* infest dogs and cats less frequently [12,13,14,15,16,17,18,19]. The Mediterranean ticks *Hyalomma marginatum marginatum* and *Hy. lusitanicum* [16,20,21] and the rodent tick *Ix. trianguliceps* [16,22] have been found to attack dogs sporadically. In the group of ticks that parasitize dogs and cats, there are two species (i.e., *Rh. sanguineus* and *Ix. canisuga*) associated with the human environment, in which their entire development cycle can be completed. A majority of ticks parasitizing companion animals can attack humans (e.g., [23,24,25]).

Companion animals travelling with their owners over long distances on the same continent or between continents are exposed to an increased risk of infestations by various species of ticks with considerable vector competence and infection with pathogens transmitted by these ticks. Most often, ticks are imported on dogs or cats in the parasitic stage. However, unattached ticks can also be found in animal fur or transport containers, and later reach the skin of other animals or their keepers

The spread of ixodid ticks by companion animals and infection of these hosts with tick-borne pathogens during tick feeding is facilitated by specific biological and physiological features distinguishing these arthropods from other bloodsucking ectoparasites.

Ticks are attached to host skin for several days or even weeks. depending on the species and developmental stage [26,27,28], and their feeding dynamics may be influenced by various factors, including the intensity of invasion [29], the presence of ticks from the same or different species co-feeding on the same host [30], the sexual structure of foraging specimens [31], and climate conditions [32].

During blood ingestion, infected ticks can introduce factors of tick-borne diseases contained in tick saliva (e.g., viruses, bacteria, and protozoa) into the host. Canine babesiosis (*Babesia* spp.), borreliosis (*Borrelia burgdorferi* s.l.), ehrlichiosis (*Ehrlichia* spp.), rickettsiosis (*Rickettsia* spp.), and anaplasmosis (*Anaplasma* spp.) [19,33,34,35,36,37,38,39] are the most common tick-borne diseases in dogs and/or cats.

Skin lesions (granuloma, alopecia) and allergic reactions [16,40,41,42,43,44,45,46,47] as well as tick paralysis caused by the neurotoxic components of tick saliva [48,49,50,51,52,53] are the direct effects of tick parasitism in companion animals. In dogs, tick bites may induce production of specific immunoglobulin E antibodies to oligosaccharide galactose-α-1, 3-galactose (anti-α-Gal antibodies), which can be a source of red meat allergy [54].

Incidents of importation of ticks to new areas by animals transported by humans have been repeatedly described in the world literature [55,56,57,58,59,60]. A majority of reports have described importation of ticks by exotic reptiles transported from endemic areas to other regions of the world [55,61,62,63,64,65,66,67]. However, the scale of this phenomenon and the potential threat to animal and human health posed by imported ticks and pathogens in the new environment have rarely been assessed.

In this study, we highlight the role of companion animals, mainly dogs, travelling with their owners or importation of these animals in the transmission of ticks and tick-borne diseases to non-endemic areas. We also emphasize the role of dogs and cats in the spread of autochthonous tick and pathogen species.

The risk of transmission of pathogen-infected ticks by dogs and cats transported by humans has increased substantially in recent decades due to the rise in tourist and economic migration rates. Most agents of tick-borne diseases (e.g., *Borrelia* spirochetes [13,68,69,70,71], *Rickettsia* [69,70,72,73,74], *Anaplasma phagocytophilum* [68,71,73], and tick-borne encephalitis virus (TBEV) [75,76]) are pathogenic to not only companion animals but also humans [25,73,77]. Companion animals and humans can also be infected with bacteria, protozoa, and helminths transmitted by other vectors (e.g., fleas, mosquitoes, and phlebotomine sand flies; [78,79,80]). Therefore, clinical and laboratory methods for diagnostics of diseases transmitted by ticks and other bloodsucking arthropods, which facilitate identification of the pathogenic factor and effective treatment, arouse increasing interest (e.g., [25,81,82,83,84,85,86,87]).

Due to the increase in the incidence of animal and human tick-borne diseases recorded worldwide, there is a need to trace the potential routes of transmission thereof to areas where they did not occur or rarely appeared previously. Determination of these routes will prompt effective measures for limiting the harmful results of tick parasitism.

## 2. Alien and Autochthonous Ticks and Tick-Borne Pathogens Carried by Travelling and Imported Companion Animals

Cases of transport of ticks and associated pathogens by companion animals have been reported in various countries, but only some countries have monitored this process. In Europe, the role of dogs in the transmission of autochthonous and exotic tick species has been assessed, specifically in Germany [88,89,90,91,92,93,94,95], the UK [17,20,96,97], Benelux countries [98,99], Sweden [100], Switzerland [101], Hungary [15,102], Cyprus [103], Greece [104], Italy [105], Spain [106], and Portugal [107]. However, there are very few reports on the role of transported cats in the transmission of ticks to other habitats [108,109,110], which is probably related to the fact that cats accompany their travelling owners less frequently than dogs. Additionally, cats usually stay indoors when they accompany their owners and are thus not exposed to contact with ticks present in the environment.

Tick infestations and transmission of pathogens in dogs and cats are supported by the presence of tick species with high epidemiological importance in urban and suburban areas that are often visited by owners of these animals. An example is the occurrence of *Rh. sanguineus, Rh. turanicus, Rh. bursa, Ix. ricinus, De. marginatus,* and *Ha. punctata* in the parks of Rome and other Italian cities [111,112,113] and the *Rh. sanguineus* group, *Ha. parva, Hy. marginatum, Rh. bursa, Ix. ricinus, De. marginatus, Hy. anatolicum, Hy. detritum, Hy. excavatum*, and *Ha. punctata* in Ankara (Turkey) [114]. The likelihood of infection of companion animals with pathogens in these areas is associated with the level of the prevalence of these microorganisms in ticks, which is higher than in natural habitats [115,116,117,118].

Dogs are usually infested by tick adult stages [15,16], less often by nymphs [15,16,106], and sporadically by larvae [16,20,22,46,119]. Dog’s head (especially the eye area, ears, and muzzle), legs, and neck are the most commonly attacked body areas [16,120,121]. The frequency of tick attachment to the skin is associated with the breed of dogs and their temperament [120]. In cats, the greatest number of ticks attach to the head (ears, chin, face, and muzzle) and the neck [121].

In the world, *Rh. sanguineus* sensu lato is the most commonly imported dog tick (e.g., [20,101,109,122,123,124]). This species is distributed in large areas in warm climate regions in both hemispheres, where it can be found in kennels, buildings, and gardens [4,125].

*Rh. sanguineus* is a common tick species in the Mediterranean region, from where specimens are most often imported on dogs to countries in the central and northern part of the continent, including the UK [109,126,127,128,129], Germany [88,92,130,131,132,133,134,135], Poland [136], the Czech Republic [137,138], the Netherlands [98,139], Denmark [140,141,142,143], Belgium [108,144], and Switzerland [145]. Less frequently, *Rh. sanguineus* ticks are transferred to European countries from other endemic areas of this species, like the Dominican Republic [88] and Sudan [140]. As shown in literature, the number of these cases clearly increased after the economic and political transformations in Europe at the end of the 20th century. This is associated with the growing interest in tourism and business trips to remote locations in Europe, Americas, Africa, and Asia, where high prevalence of ticks from the *Rh. sanguineus* group in dogs from urban and rural environments is observed. For instance, *Rh. sanguineus* sensu lato (s.l.) has been found to attack 63.6% of autochthonous dogs in Italy [146], 53% in Spain [106], 51.32% in Northern Algeria [147], 59.6% on the Mexico-USA border [148], 80% in north-central Nigeria [149], 80% in north-eastern Thailand [150], and as many as 98.33% in Punjab, Pakistan [151]. Similarly, in the endemic areas of the *Rh. sanguineus* group (*Rh. sanguineus* sensu stricto, and *Rh. pusillus*) in southern Italy, these species most often infest cats [152].

Travelling and imported dogs have been shown to be infested by the largest number of adults and nymphs but only single larvae of *Rh. sanguineus* and other tick species [20]. An unusual case was the transfer of approximately 50 or more mobile engorged and unfed *Rh. sanguineus* larvae infesting a five-old Jack Russel, which was noticed 18 h after the dog had returned from Greece [153].

The importation of *Rh. sangiuneus* s.l. ticks on animals to non-endemic areas has considerable importance for public health, as they are vectors and/or potential reservoirs of numerous pathogens. These include *Ehrlichia canis, Anaplasma platys*, and *A. phagocytophilum,* protozoa *Babesia vogeli, B. canis, Babesia vulpes*, and *Hepatozoon canis*, zoonotic *Rickettsia* species (*Rickettsia conorii* complex and *R. massiliae*), and the filarioid nematode *Cercopithifilaria bainae* [154,155,156,157,158,159,160]. Besides *Rh. sanguineus*, other alien tick species were occasionally transported by dogs to various European countries (Table 1 and Table 2).

Some of these tick species can live in new habitats with favourable conditions. Engorged females lay eggs, and juvenile stages (larvae and nymphs) transform into subsequent developmental stages. Hungry active stages of these ticks can attack hosts present in the surroundings [88,161]. The introduction of some species (*Rh. sanguineus, Hy. marginatum*, *Hy. suspense*) to non-endemic areas is possible due to the climate and environmental changes occurring worldwide (e.g., [145,162,163]).

An interesting case of introduction of *Rh. sanguineus* to Poland and mass reproduction of this tick in an apartment in Warsaw was described by Szymański [136]. These ticks from a dog travelling with an Italian family infested the dog of the Polish family when they spent holidays together in the Mazury region. After return to Warsaw, an engorged *Rh. sanguineus* female detached from the host skin and laid eggs, from which larvae developed. *Rh. sanguineus* ticks can complete their full life cycle indoors. Cases of household *Rh. sanguineus* infestations have been reported from other countries as well, including Germany [88], England [96,97,164], and France [165].

In Europe, dogs and cats most frequently transfer autochthonous species of ticks, which are vectors of numerous animal tick-borne diseases. For instance, a group of 2373 ticks removed from dogs and cats in Belgium was dominated by the castor bean tick *Ix. ricinus* (76.4%) and the European hedgehog tick *Ix. hexagonus* (22.6%); single specimens of the meadow tick *De. reticulatus* (0.8%) were found as well. *Rh. sanguineus* specimens, which accounted for 0.3% of the total number, were found on dogs after return from endemic areas of this tick species [108].

In the UK in 2005–2016, *Rh. sanguineus* (46.2%) and *I. ricinus* (33.8%) ticks were the most common species of ticks found on animals with a history of travel mainly to southern Europe, the USA, and the United Arab Emirates [20]. In Spain, *Rh. sanguineus* s.l. (53%) was the most abundant autochthonous species in a group of 1628 collected adult ticks. Other ticks accounted for a lower percentage: *De. reticulatus* (9%), *Ix. ricinus* (9%), and *Ix. hexagonus* (4%). In a group of 660 dogs sampled in 26 veterinary clinics, as many as 507 dogs (76.8%) were infested by at least one adult tick [86]. In Greece, the majority of ticks infesting 150 dogs (48.4%) were *Rh. sanguineus* s.l. (70.1%), whereas other species were less numerous (i.e., *Ha. parva* (14.7%), *Rh. turanicus* (11.4%), and *Ha. concinna* (2.4%)). A total of 11.1% of 344 sampled ticks were vectors of at least one microorganism (5.5%: *Cercopithifilaria bainae*, 2.9%: *Hepatozoon canis*, 1.7%: *Rickettsia hoogstraalii*, 1.2%: *Hepatozoon felis*, 0.6%: *Rickettsia massiliae*, 0.6%: *Theileria ovis*, 0.3%: *Anaplasma platys*, and 0.3%: *Coxiella* like-endosymbiont) [166].

*Rh. sanguineus* transferred on travelling and imported dogs into non-endemic areas may pose a health risk by transmission of pathogens (e.g., *Ehrlichia canis)* to native dogs [167,168]. Canine hepatozoonosis caused by the apicomplexan protozoan *Hepatozon canis* was diagnosed in three dogs imported to the United Kingdom from Mediterranean countries [169]. The infection by pathogens acquired by animals in the travel destination area is often confirmed by serological tests only after returning home [170]. Dogs are a reservoir of infectious agents of human diseases, like Mediterranean spotted fever caused by *Rickettsia conorii* [171,172].

Various microorganisms were detected in *Rh. sanguineus* ticks carried into new areas on dogs, including *Ri. conorii* [173,174], *Ri. massiliae*/Bar29, and *Coxiella* sp. [175]. Contact with dogs or cats infested by pathogen-infected *Rh. sanguineus* ticks increases the likelihood of the spread of tick-borne pathogens, like spotted fever group (SFG) rickettsiae. Symptoms of spotted fever group (SFG) rickettsiosis were confirmed in members of a family from south France living in a house where 22 *Rh. sanguineus* ticks were collected from the floor behind furniture. Of these ticks, 20 were infected with *Rickettsia*; more specifically, nine and seven specimens were infected by *Ri. conorii subsp. caspia* and Ri. massiliae, respectively, whereas both rickettsiae were identified in four ticks [165]. As shown by Edouard et al. [176], some patients in France were diagnosed with rickettsiosis caused by *Ri. sibrica mongolitimonae,* another representative of the spotted fever group. *Ri. massiliae* and *Ri. sibirica mongolitimonae* were detected in *Rh. sanguineus* and *Rh. pusillus* ticks sampled from their dogs and cats, respectively. *Rh. pusillus* ticks infected by *Ri. sibirica mongolitimonae* were also collected from cats’ litter boxes and beds. There are reports of cases of human infection with *Ri. conorii* contracted during removal of ticks from dogs [177].

An especially high risk of development of tick-borne and other arthropod-borne diseases in companion animals has been observed in attractive tourist regions characterized by a high prevalence of pathogens transmitted by ticks and other arthropods (e.g., [79,178,179,180,181,182,183,184,185]). For instance, it was reported that the prevalence of *Ri. conorii* in dogs in southern Italy ranged from 72% to 73.60%. The prevalence of *An. phagocytophilum* was in the range from 32.80% to 38%, *B. canis* from 5.17% do 70%, and *Eh. canis* from 21.70% to 46%. The prevalence of *Co. burnetii* was estimated at 31.50% [181,186].

In the north of Spain, antibodies against these pathogens were detected in 11.33% of tested dogs, with prevalence of 1.26% for *Anaplasma* spp., 0.9% for *Eh. canis*, and 0.72% for *Bo. burgdorferi* transmitted by ticks and 8.99% for *Le. infantum* and 0.18% for *Di. immitis* transmitted by sand flies and culicid mosquitoes, respectively [187].

## 3. Tick Preventive Behaviors and Practices of Dog and Cat Owners

The effects of tick parasitism on companion animals and their owners can be limited by application of various methods for protection against tick attacks recommended by the Centers for Disease Control and Prevention [188] and the European Scientific Counsel Companion Animal Parasites [189].

The risk of human and animal contact with ticks can be minimized by avoidance of habitats with high numbers of ticks at the peak of their seasonal and diurnal activity. Another important element of tick prophylaxis is the preventive behavior of animal owners outdoors (e.g., limitation of contact with plants, avoidance of sitting on grass and under shrubs, wearing protective clothes to prevent ticks from attachment to the skin, inspection of clothes or skin to find ticks), and removal of the feeding tick from the skin as soon as possible after attachment. Tick attacks can be limited with effective repellents (e.g., [190]). As shown in various studies, inspection of the body on return home, wearing protective clothes, and the use of repelents are the most preferred methods used by humans for personal protection against tick attachment to the skin [191,192,193,194,195,196,197].

Owners of dogs and cats are advised to inspect the hair and skin of their animals, especially the head, ears, shoulders, and upper leg areas, when the animals stay outdoors during the tick activity period. They should also use various acaricidal and/or repellent substances contained in numerous formulations available on the market (e.g., spot-on, pour-on, baths, and insecticide-impregnated collars).

Orally administered parasiticides against ticks are also effective in protecting dogs against ticks and tick-borne pathogens (e.g., [198,199,200,201,202]). A single oral dose reduced the survival rate of over 98% of ticks present on the host 48 h after treatment and effectively protected against new infestations for several weeks [198,199].

Specimens attached to host’s skin are removed with various methods described in various publications (e.g., [203,204]).

## 4. Conclusions

The most important role in rapid transmission of ticks and associated pathogens over long distances is ascribed to seasonally migrating birds (e.g., [205,206,207,208,209,210]). Nevertheless, it seems that animals accompanying their owners during foreign and domestic journeys or animals imported from other geographic regions may contribute considerably as well. The problem has to be emphasised to draw attention of owners of dogs and cats as well as veterinary staff, due to the dramatic increase in the prevalence of tick-borne diseases in these animals (e.g., [19,79,211,212,213,214,215,216,217]).

Before travelling to endemic areas of tick-borne diseases, tourists should be acquainted with prophylaxis methods to protect themselves and their companion animals against tick attacks. Information about tick species that infest dogs and cats most frequently, periods of the highest tick questing activity, and threats of zoonotic tick-borne diseases posed to animal health is indispensable for owners of companion animals.

## Figures and Tables

**Table 1:** Examples of alien ticks imported on dogs and cats into non-endemic areas in Europe.

Tick Species	Areas of Tick Infestations of Dogs or Cats	Place of Import of Ticks	Source of Information Authors, [Ref.]
***Ixodes holocyclus***	Australia	United Kingdom (UK)	Adamantos et al. [49]
***Ixodes pacificus***	USA	UK	Jameson et al. [109]
***Amblyomma americanum***	USA	UK	Jameson et al. [109]
***Dermacentor variabilis***	USA	UK	Abdullah et al. [17]; Jameson et al. [109]
***Dermacentor albipictus***	USA	UK	Jameson et al. [109]
***Haemaphysalis leachi***	South Africa, Tanzania, Zambia	UK	Dutto and Selmi [16]
	Rhodesia (Zimbabwe), South Africa, Tanzania, Zambia	UK	Jameson et al. [109]
***Haemaphysalis elliptica***	South Africa	UK	Hansford et al. [123]
***Hyalomma lusitanicum***	Portugal	UK	Hansford et al. [20]
***Rhipicephalus sanguineus***	Sudan	Denmark	Winding and Haarløv [140]
	Zambia	Denmark	Haarløv [141]
	No data	Denmark	Winding et al. [142]
	No data	Denmark	Willeberg [143]
	Spain, Dominican Republic, Italy, France, Greece	Germany	Dongus et al. [88]
	No data	Belgium	Claereboutt et al. [108]
	No data	UK	Hansford et al. [123]
	Cyprus, Spain	UK	Hansford et al. [123]
	Africa, Australia, Canada, Iran, Malta, Philipines, Singapore, West Indies, Saudi Arabia, South Africa, Mideterranean region (Spain, Cyprus, Tunisia), USA	UK	Jameson et al. [109]
	Greece	UK (London)	Wright et al. [121]
Haemaphysalis leachi *	Africa	UK	Jameson et al. [109]

* ticks imported on cats.

**Table 2:** Examples of autochthonous tick species carried by imported and travelling dogs in Europe

Tick Species	Areas of Tick Infestations of Dogs	Place of Import of Ticks	Source of information Authors, [Ref.]
***Ixodes ricinus***	Belgium, The Netherlands, Germany Finland	United Kingdom (UK) UK	Jameson et al. [109] Hansford et al. [123]
***Ixodes hexagonus***	Germany France	UK UK	Jameson et al. [109] Hansford et al. [123]
***Ixodes canisuga***	France	UK	Hansford et al. [123]
***Ixodes ventalloi***	France (Montpellier)	Northwest Italy	Dutton and Selmi [16]
***Dermacentor reticulatus***	France, Bulgaria	UK	Hansford et al. [123]
